# Linear Temporal Logic (LTL) Based Monitoring of Smart Manufacturing Systems

**Published:** 2015

**Authors:** Gerald Heddy, Umer Huzaifa, Peter Beling, Yacov Haimes, Jeremy Marvel, Brian Weiss, Amy LaViers

**Affiliations:** 1 University of Virginia, Charlottesville, VA, 22904, USA; 2 University of Virginia, Charlottesville, VA, 22904, USA; 3 University of Virginia, Charlottesville, VA, 22904, USA; 4 University of Virginia, Charlottesville, VA, 22904, USA; 5 National Institute of Standards and Technology, Gaithersburg, MD, 20899, USA; 6 National Institute of Standards and Technology, Gaithersburg, MD, 20899, USA; 7 University of Virginia, Charlottesville, VA, 22904, USA

## Abstract

The vision of Smart Manufacturing Systems (SMS) includes collaborative robots that can adapt to a range of scenarios. This vision requires a classification of multiple system behaviors, or sequences of movement, that can achieve the same high-level tasks. Likewise, this vision presents unique challenges regarding the management of environmental variables in concert with discrete, logic-based programming. Overcoming these challenges requires targeted performance and health monitoring of both the logical controller and the physical components of the robotic system. Prognostics and health management (PHM) defines a field of techniques and methods that enable condition-monitoring, diagnostics, and prognostics of physical elements, functional processes, overall systems, etc. PHM is warranted in this effort given that the controller is vulnerable to program changes, which propagate in unexpected ways, logical runtime exceptions, sensor failure, and even bit rot. The physical component’s health is affected by the wear and tear experienced by machines constantly in motion. The controller’s source of faults is inherently discrete, while the latter occurs in a manner that builds up continuously over time. Such a disconnect poses unique challenges for PHM. This paper presents a robotic monitoring system that captures and resolves this disconnect. This effort leverages supervisory robotic control and model checking with linear temporal logic (LTL), presenting them as a novel monitoring system for PHM. This methodology has been demonstrated in a MATLAB-based simulator for an industry inspired use-case in the context of PHM. Future work will use the methodology to develop adaptive, intelligent control strategies to evenly distribute wear on the joints of the robotic arms, maximizing the life of the system.

## 1. Introduction

Industries active in the manufacturing sector exist in a competitive landscape where profitability is heavily influenced by their operational directives. A manufacturer choosing to implement Smart Manufacturing Systems (SMS) would likely drive down their costs, improve their manufacturing goals, and meet continuous improvement objectives. Robotics and automation are often a logical and feasible ingredient to increasing productivity, while also maintaining or improving product quality and operational safety goals. A recent national report on advanced manufacturing showed that industry use of automation positively impacted profitability such that manufacturers were more likely to keep their internal operations vertically integrated (Anderson, 2011). This report also highlights the important role that next-generation robotics will play in the future of manufacturing such as realizing improvements in flexibility, time to market, cost, quality, and human safety.

Prognostics and Health Management (PHM) is a comprehensive field that attempts to create the systems and methods which manufacturers employ to enhance their asset maintenance programs. PHM standards are developed as a better alternative to traditional reactive maintenance programs primarily defined by initiating action only after a breakdown or some lost production time event has occurred. It is through the use of condition-monitoring, diagnostic, and prognostic methods that PHM attempts to understand the states of the system and create a manufacturing environment where maintenance is carried out on a more preventative, predictive, and proactive basis as compared to being purely reactive. A PHM approach to maintenance proves beneficial by reducing manufacturer dependence on non-value added maintenance time and capital of parts replacement. PHM strives to increase asset lifespan while operating at lower cost.

The emergent contributions of robots to higher efficiency and product quality in smart manufacturing processes have also introduced new sources of risk thereto including: (i) safety risks resulting from the collaborative and proximal interface between humans and robots; (ii) maintenance schedule and operations; and (iii) sensitivity to irregularities associated with out-sourced parts and raw materials, among others. In this sense, the centrality of PHM in smart manufacturing has necessitated expansion to embrace systems-based risk modeling, assessment, management, and communication (Haimes, 2012, 2005). In particular, the interdependencies between the robotics subsystems and the human operators necessitate an understanding of the epistemological human behavior and responses under extreme events originating from either the robotics or human subsystems.

It is then necessary to think about PHM in the context of robotics as both of these fields (PHM and robotics) enable development of SMS. As private and public investment rises to implement and develop next-generation robotics, we will also need to create the high-level control strategies which seek to attain condition-based PHM goals. This work introduces a novel robotic monitoring system as a step towards PHM with the motivation to display and predict both discrete system failures and continuous motion wear.

After further review of SMS, the paper introduces an industry-inspired use case. We will then apply a novel methodology from (Huzaifa, Umer and Marvel, Jeremy A. and LaViers, Amy E., 2015) that can incorporate a high-level description of the correct behavior for the robotic system to our use-case. This is accomplished with linear temporal logic (LTL) specifications and a labeled, discrete representation of the SMS. By generating a Büchi automaton representation of the high-level specification phrased in LTL, the system dynamic and correct behavior can be represented in the same product automaton. This resulting automaton encodes all system behavior that is within the specification and forms the basis of the monitoring system. This methodology has been implemented in a MATLAB-based simulator, which also tracks a continuous system variable.

Finally, the paper presents results of this methodology with respect to PHM. Correct control sequencing is represented at a high-level using task-level labels for the discrete system model. It is over these task-level labels that the specification will monitor the correct behavior of the system. Wear monitoring is achieved using a differential equation model of wear in both loaded and unloaded conditions. These discrete and continuous statuses are tracked and displayed and will be used to develop corrective control strategies to maximize the lifetime of the robotic system. This work is part of a larger effort to create a modular, adaptive multi-scale PHM scheme (AM-PHM) where we take operational demand profiles, generate performance and health assessments, then create operational objectives.

## 2. Prognostics and Health Management for Smart Manufacturing Systems

Prognostics and health management (PHM) technologies reduce time and costs for maintenance of products or processes through efficient and cost-effective diagnostic and prognostic activities. In 2010, a comprehensive review was conducted of prognostic and diagnostic methodologies for condition-based maintenance (CBM) that presented the existing strategies within four categories: physical models, knowledge-based models, data-driven models, and combination (hybrid) models (Peng, Dong, & Zuo, 2010). This review highlighted many specific methods across these four categories (e.g., Hidden Markov Models, Bayesian network-related methods, Fuzzy Logic, Principal Components Analysis) along with their successes and limitations. No one method stood out as being sufficient to provide both diagnostic and prognostic intelligence at multiple levels. This review demonstrated that for every method’s strength, there was at least a single weakness. Similarly, another review of existing methods was conducted in 2012 that focused on comparing time-based maintenance (TBM) and condition-based maintenance (CBM) (Ahmad & Kamaruddin, 2012). TBM, commonly referred to as preventative maintenance, is typically simpler to implement (in that maintenance is scheduled based upon a specific unit of time; e.g., cycle time) while CBM, sometimes termed predictive maintenance, may ultimately be more cost effective if a process’s or equipment’s health data accurately reflects its current state and allows a machine to run longer until maintenance (as compared to a TBM schedule). The challenge in CBM is gathering sufficient data to make a reasonably accurate prediction. Both of these studies revealed that PHM is applicable to both products and processes; this makes PHM a tremendous, and necessary, asset to SMS.

Product PHM (providing health monitoring, diagnostics, and/or prognostics for a finished system; e.g., automobile, aircraft, power generation station) is more widespread as compared to process PHM (providing health monitoring, diagnostics, and/or prognostics to a system that integrates one or more pieces of equipment to complete a task; e.g., assembly process, welding process, machining process). (Batzel & Swanson, 2009) (Holland, Barajas, Salman, & Zhang, 2010) (Hu & Koren, 1997) (Shen, Wan, Cui, & Song, 2010). Likewise, PHM techniques have been developed and applied more widely at the component/equipment level, yet some work has occurred at the higher/system levels. For example, innovative methods have been developed for various machining operations (Al-Habaibeh & Gindy, 2000) (Altintas, Verl, Brecher, Uriarte, & Pritschow, 2011) (Biehl, Staufenbiel, Recknagel, Denkena, & Bertram, n.d.) (Borisov, Fletcher, Longstaff, & Myers, 2013). System level PHM methods have also been developed, yet seem to be very focused in their applicability and/or limited in capability (Barajas & Srinivasa, 2008) (Datta, Jize, Maclise, & Goggin, 2004) (Hofmeister, Wagoner, & Goodman, 2013).

The paper (Vogl, Weiss, & Donmez, 2014) conducted a detailed review of existing standards that were designed to help guide implementation of PHM in manufacturing. Specifically, many of the current PHM standards were developed within the International Organization for Standardization (ISO) and focus primarily on condition monitoring and diagnostics (ISO, 2002) (ISO, 2003) (ISO, 2012). Few standards include discussion of prognostics (ISO, 2004). The standards review highlighted that only very specific processes benefited from these standards; they are not considered broadly applicable. This study highlights a gap in that no standards are currently available that are both robust and flexible to address the diverse and dynamic environments presented by Smart Manufacturing.

Smart Manufacturing presents a paradigm shift in that manufacturers are thinking differently about how they implement their production technologies, tools, and teams. The field of robotics has already released and is actively working towards a next generation of new products, bolstered by developments in low-level controllers such as proximity detection, image processing, and precise human-safe actuators. In addition, collaborative robotics systems are emerging, enabling robots to work side-by-side with humans and other robots without requiring physical safety barriers. Collaborative robotics are characterized by:

1. Lower total implementation costs
2. Reduced barrier-to-entry in the form of operational technical skill
3. Improved efficiencies and overall equipment effectiveness (OEE) as discussed in (Jeong & Phillips, 2001)
4. Flexible spatial feasibility and responsive configurations
5. Increased safety features allowing humans to work alongside them

For many small and medium-sized manufacturers, the cost of integrating a robot into a historically manual process is the most prohibitive barrier to automation. While the purchase price of a robot is sometimes significant, it is dwarfed by the cost of process integration, programming, and support. Many collaborative robot technologies effectively reduce the overhead associated with safety, programming, and factory floor real estate. As such, the promise of reduced cost and ease of use are seen as a means by which even small and medium-sized enterprises may access and adopt automation technologies (Marvel, 2014).

However, with safety being the principal focus for the current development of collaborative technologies, system performance and reliability have yet to be verified. As such, these systems require the means by which end users can guarantee the application performance, and ultimately establish confidence in the systems on which they will rely. Proper health monitoring and prognostics modeling of system and process performance, in particular, will provide end users with the necessary insights into the reliability of such emerging smart manufacturing technologies.

With this profound interest for installing robotic and other automated platforms, it is increasingly important to create the high-level control strategies necessary for operating them. The competitive landscape has changed the way corporations manage their supply chain solutions. A plant manager cannot lead his or her world class facility with only reactive maintenance systems in place. Rather, PHM based techniques could be seen as a corollary to the cultural principles established in Total Productive Maintenance (TPM) (Nakajima, 1988) and Lean Manufacturing (Shah & Ward, 2003).

## 3. The Industry-inspired Use-Case

For our use-case, we have created a scenario with two robots collaborating together to accomplish a task in a work cell that is assumed to be a part of an entire production line. The task to be completed can be subdivided into a pick and place operation combined with a drilling operation, as seen later in Figure 3. The pick and place will be performed by a robot which we will name “Ben”. The drilling operation is performed by a robot named “Mike”.

Boxes are generated according to a predetermined cycle time, arriving from an upstream work cell and appearing on a conveyor in front of Ben. Ben picks up a single box after it has been detected, rotates his torso actuators ninety degrees, and places it on a second conveyor that is elevated off the factory floor. Boxes then continue their conveyance route, already facing the correct orientation to receive the drilling operation. When a box is detected in front of Mike, the end effector is extended, grabs the box, drills a hole, and retracts the arm.

We will engage the use case to show the many motion trajectories that could be employed to accomplish this specific work cell’s task. It is an exciting contribution of the work to introduce the notion that we can generate redundant motion sequences to be leveraged for PHM. These will later be identified by the novel monitoring methodology achieved by a formalized separation between the overall system task and the single strategy employed at any one point in time.

It should be noted the use-case assumes a dynamic model of wear that shows increases in wear over time as the number of movements increase in the robot. We are also using a discrete transition system model of each robot’s behavior and capabilities.

## 4. An LTL-Based Monitoring Simulator For The Industrial Use-Case

We will now review the individual components of the software simulator framework as implemented on the industry inspired use case. This includes the representation of the involved robot subsystems as discrete transition systems. Further, we explain the linear temporal logic based high level objective description and monitoring.

### 4.1. Transition System Representation

**Figure 1:**
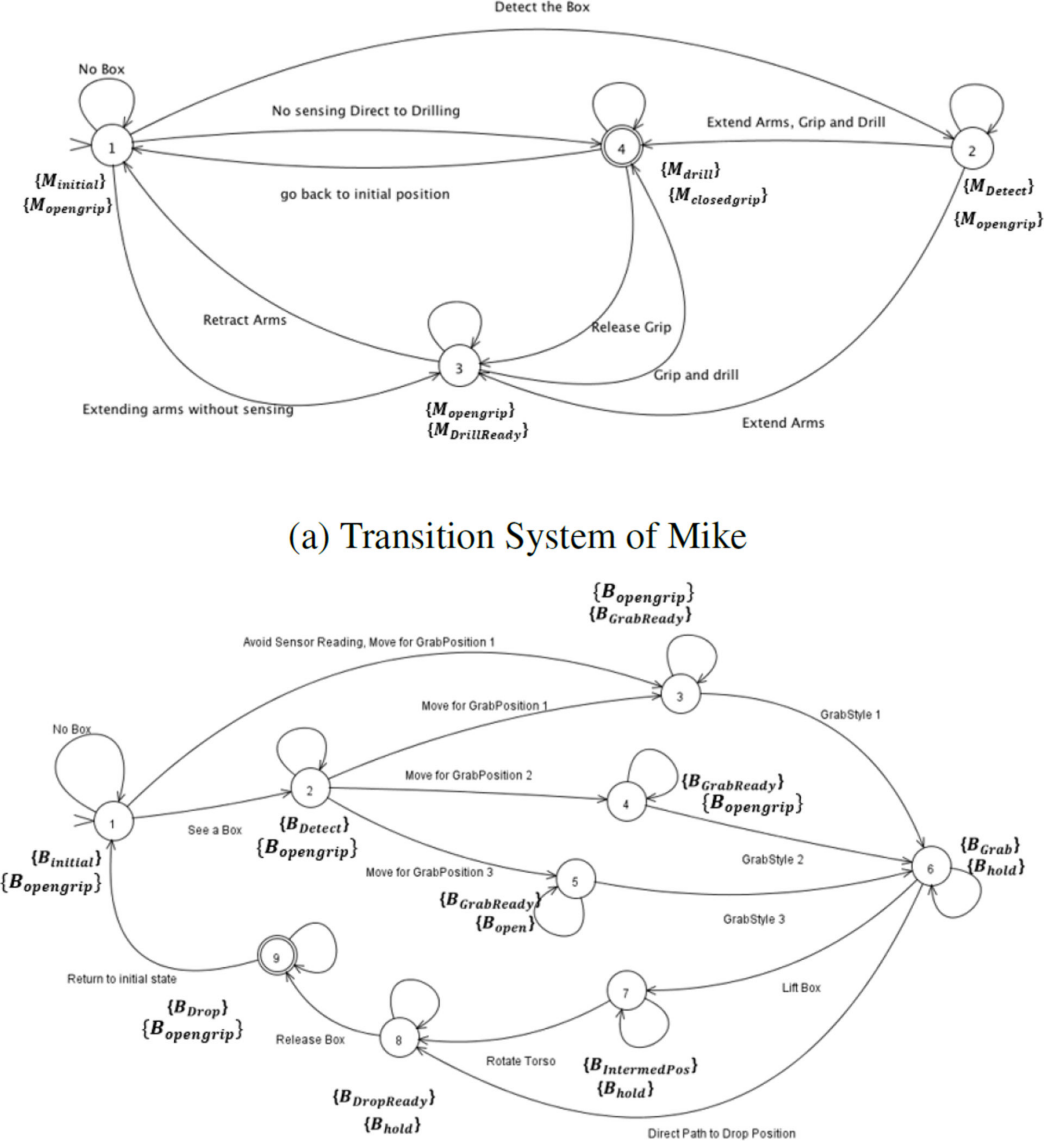
Transition Systems of the Robots.

The two robots in our use case are represented in the form of discrete transition systems. A discrete transition system is a well known concept in Computer Science where it is extensively used in formal proofs for different algorithms and software. For our case, we have also incorporated a continuous state variable in the respective transition systems for representing the wear in the robots. The transition systems of the robots for the industry inspired use-case are given in the Fig. 1. Using notation described in (LaViers, Chen, Belta, & Egerstedt, 2011), for the two robots this representation is given as:

(1) $$T_{1}=({𝒬}_{1},q_{0_{1}},\to_{1},\Pi_{1},h_{1},C_{1},w_{1})$$

(2) $$T_{2}=({𝒬}_{2},q_{0_{2}},\to_{1},\Pi_{2},h_{2},C_{2},w_{2})$$

*T*_1_ represents transition system for Mike and *T*_2_ represents transition system for Ben where

- ${𝒬}_{1}=\{q_{1}^{1},q_{2}^{1},q_{3}^{1},q_{4}^{1}\}$ is the finite set of Mike’s states, either hand labeled by a user or generated automatically through a segmentation framework. ${𝒬}_{2}=\{q_{1}^{1},\ldots ,q_{8}^{2},q_{9}^{2}\}$ is the similar set of Ben’s states. Superscripts indicate the robot (1 is for Mike, 2 is for Ben).
- *q*_0_1__ and *q*_0_2__ are the initial states of Mike and Ben respectively;
- →_*i*_ ⊆ 𝒬_*i*_ × 𝒬_*i*_ is a reflexive transition relation of Mike (if *i* = 1) or Ben (if *i* = 2) , where each state has a self-loop, allowing for one robot to transition to a new state without that requirement being imposed on the other robot;
- Π_1_ = {*M_initial_, M_opengrip_, M_detect_, M_drillready_, M_drill_, M_closedgrip_*} is a finite set of atomic propositions for robot Mike.
  Similarly, Π_2_ = {*B_initial_, B_opengrip_, B_Detect_, B_Drop_, B_grabReady_, B_Grab_, B_hold_, B_IntermedPos_, B_DropReady_*} is a finite set of propositions for Ben.
  These propositions represent the status of different subtasks performed by Mike and Ben respectively;
- *h_i_* : 𝒬_*i*_ ↦ 2^Π_*i*_^ is a satisfaction (output) map, where state $q_{j}^{i}$ satisfies the set $h_{i}(q_{j}^{i})$ of propositions from Π_*i*_. 2^Π_*i*_^ represents a set of all possible combinations of propositions of one robot. Thus, *h_i_* is a mapping of these combinations to each one of the states in the robot *i*. It can be seen in Fig. 1 how each of the states has a combination of individual propositions;
- *C*_1_ and *C*_2_ are sets of pairs of the form (*f*(*x, t*), τ). For *C*_1_ we have $\left(f_{1}^{1}(x,t),\tau_{1}^{1}),\ldots ,(f_{n\times r\times e}(x,t),\tau_{n\times r\times e}\right)$ such that $\left(f_{j}^{1}(x,t\right)$ represents dynamics of a continuous parameter for duration of $\tau_{j}^{1}$. In the final pair, *n* = 6 and defines the number of degrees of freedom in Mike; *r* = 13 and is the number of motion primitives in Mike; *e* = 2 representing the two environmental cases e.g., loaded and unloaded condition, for Mike. Similarly for *C*_2_ we have $\left(f_{1}^{2}(x,t),\tau_{1}^{2}),\ldots ,(f_{9\times 13\times 2}^{2}(x,t),\tau_{9\times 13\times 2}^{2}\right)$;
- *w*_1_ : →_1_ ↦ *C*_1_ and *w*_2_ : →_2_ ↦ *C*_2_. *w*_1_ and *w*_2_ are mapping from each transition for a respective robot to a pair in corresponding *C*_1_ and *C*_2_. More simply, it is a function that maps all the transitions of a robot to a corresponding wear dynamic.

The states correspond to the robot states while performing the tasks. For example, a state can be the idle state when the robot is waiting for the sensor to detect the box in front of it. The atomic propositions represent statements about the states of the robot and they can be either true or false. The linear temporal logic (LTL) specifications, as will be explained in the next subsection, are described in terms of these statements and the system evolves in terms of them.

The next task is to combine the representation of different robots to describe the whole system in terms of a single transition system. This can be achieved using the composition of the two transition systems. This composition is achieved by taking synchronous product of the transition systems for the individual robots.

The synchronous product of two transition systems *T*_1_ and *T*_2_, denoted as *T_p_* = *T*_1_ ⊗ *T*_2_, is a new transition system with (𝒬_*P*_, *q*_0_*P*__, →_*P*_, Π_*P*_, *h_P_*). The states are Cartesian pairs of the single robot states, i.e., 𝒬_*P*_ ⊆ 𝒬_1_ × 𝒬_2_, likewise *q*_0_*P*__ = (*q*_0_1__, *q*_0_2__). Transitions exist between these joint states if and only if a transition existed between both single states, i.e., →_*P*_⊆ 𝒬_*P*_ × 𝒬_*P*_ is defined by (*q, q*′) ∈→_*P*_ if and only if *q* ≠ *q*′, $(q_{1},q_{1}^{\prime })\in \to_{1}$ and $(q_{2},q_{2}^{\prime })\in \to_{2}$, where *q* = (*q*_1_, *q*_2_) and $q\prime =(q_{1}^{\prime },q_{2}^{\prime })$. The set containing atomic propositions for the composition of the two transition systems, denoted as Π_*P*_, is a union of the individual sets of propositions for the two robots that extends to include propositions which apply to situations where both robots are active.

Now we have the transition system for the two robots defined. With a formal representation of the robots, we can now define high level tasks for the robots in terms of the states. This is accomplished with LTL specifications and their representation in the form of Büchi automaton. Next we describe the LTL based specifications.

### 4.2. Linear Temporal Logic (LTL) Specifications

What we want is a tailored transition system according to the high level objectives. This is where the LTL specifications come in. A brief introduction of the LTL operators is given as follows:

LTL formulas are described in terms of the set Π of atomic propositions. LTL specifications describe the high level objectives in the form of Boolean and temporal operators. The Boolean logic operators, that have been used, include, ¬ (negation), ∨ (disjunction), ∧ (conjunction), and → (implication). The temporal operators include, **X** (next), 𝒰 (until), **F** (eventually), and **G** (always). LTL formulas are defined over infinite words generated by the transition systems. In particular, the LTL specifications we define, describe the possible actions of our system of robots, 𝒯_*p*_.

An LTL formula ϕ is said to satisfy a word of the transition system if the formula ϕ is true for the first position of the word; **X**ϕ states that at the next state, an LTL formula ϕ is true; **F**ϕ means that the LTL formula ϕ eventually becomes true at some position of the word; **G**ϕ means that the LTL formula ϕ is true for all the positions of the word; ϕ_1_ 𝒰ϕ_2_ means ϕ_2_ eventually becomes true at some position in the word and ϕ_1_ is true until that position of the word comes. More complex and sophisticated specifications can be designed using a combination of Boolean and temporal operators. For details (Clarke, Peled, & Grumberg, 1999) can be consulted.

As an example, some high level objectives and their LTL representations are given below. We will only show the basic LTL form **G**(*Proposition*_1_ → *Proposition*_2_), as this will be the most common form used in practice by manufacturers in specifying their high level objectives.

- Ben! Stay in initial position when Mike is performing drilling
  **G** (*M_drill_* → *B_initial_*)
- Mike! do not grip unless you are in the drilling position
  **G** (*M_closedgrip_* → *M_drill_*)
- Ben! do not open your hand while you are holding the box
  **G** (¬*B_hold_* → *B_open_*)
- Mike! Stay in initial position when Ben is dropping the box
  **G** (*B_Drop_* → *M_initial_*)

**Figure 2:**
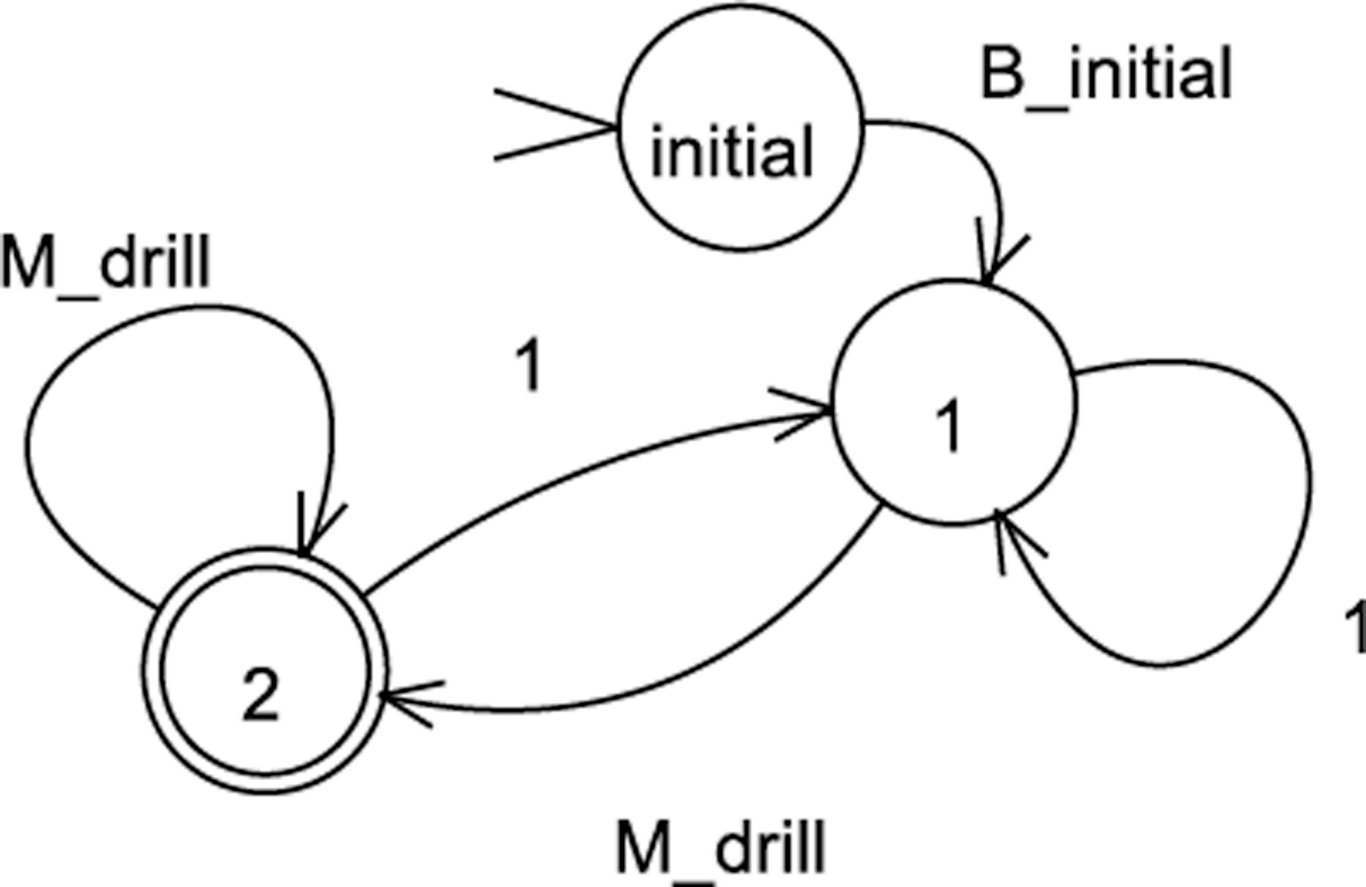
Büchi Automaton representation of an LTL specification

To check whether all words of the transition system, *T_p_*, satisfy an LTL formula ϕ over the set of propositions Π_*P*_, we need to have Büchi Automaton that accepts only the words satisfying ϕ. By the help of a tool, LTL2BA (Gastin & Oddoux, 2001), we are able to get a Büchi Automaton ℬ_ϕ_ from the LTL specification ϕ. For example, the first specification can be given in the Büchi Automaton form as pictured in Fig. 2.

A tailored representation of the system can then be had by taking a product of the system transition system *T_p_* and ℬ_ϕ_ to get the final automaton 𝒜. Now this automaton as mentioned earlier represents all the *allowed* transitions between states of the system in light of the specifications defined in ϕ. The LTL specifications are defined in such a way that they define the desired behavior of the whole system. We monitor the behavior of the system by monitoring the transitions in the system. If an error occurs, because of a sensor failure, robot motor failure etc., these specifications are not satisfied and the monitoring system returns a sequence that is **not** satisfied by *T_P_* × ℬ_ϕ_. We monitor and verify the desired movements of the robots based on the allowed transitions by using an interface between MATLAB and VREP.

## 5. Applications To PHM

Through the use of LTL we are able to build the discrete sensor oriented piece of the monitoring scheme. The transition system’s representation of the continuous parameter for each robot, *C*_1_ and *C*_2_, allows us to track differential wear functions over time. The two of these combine to create the complete system monitor for use in PHM.

### 5.1. Results of the LTL-Based Monitor

**Figure 3:**
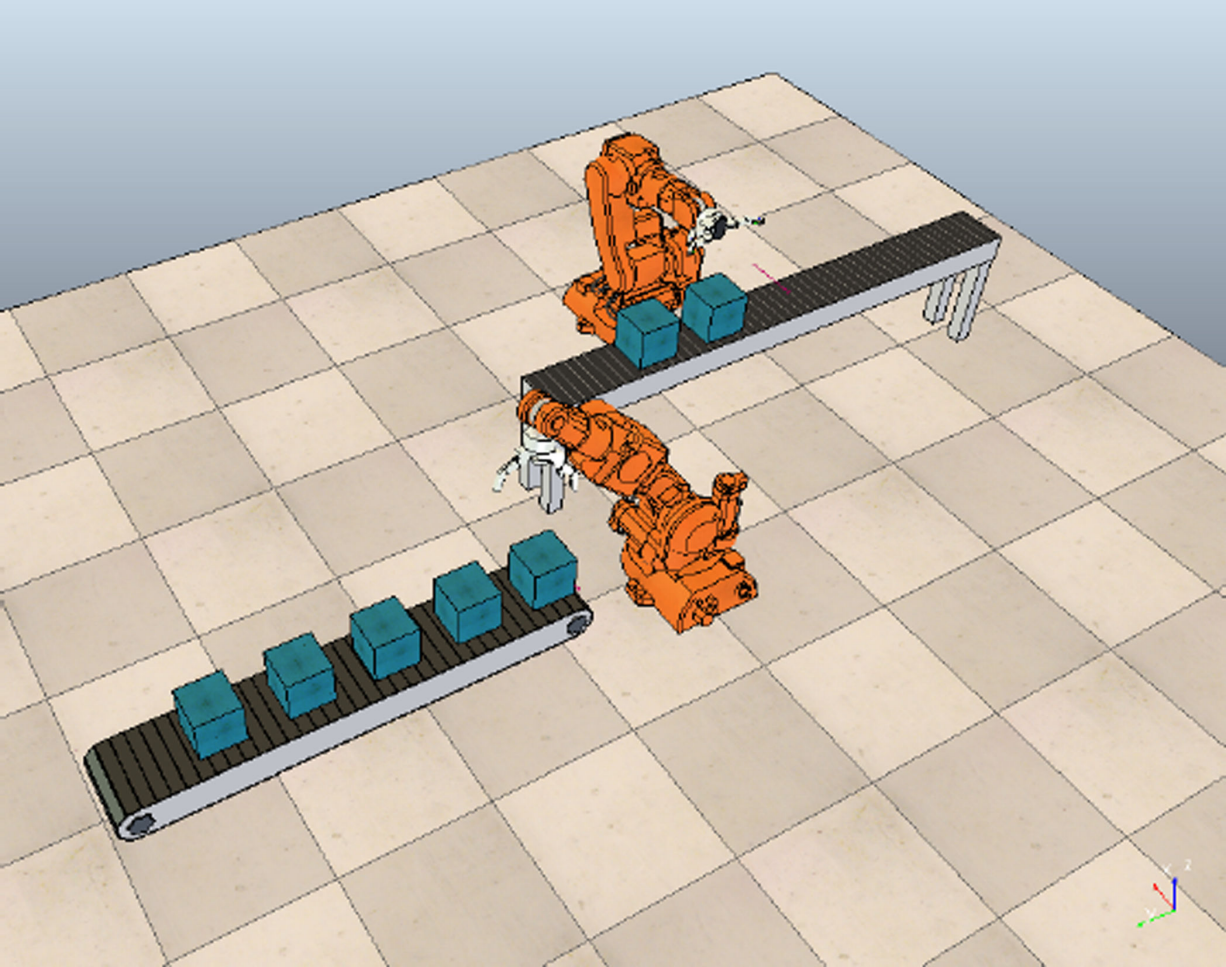
VREP simulation environment of the use case complete with two robots performing the pick and place of the box and subsequent drilling operation.

**Figure 4:**
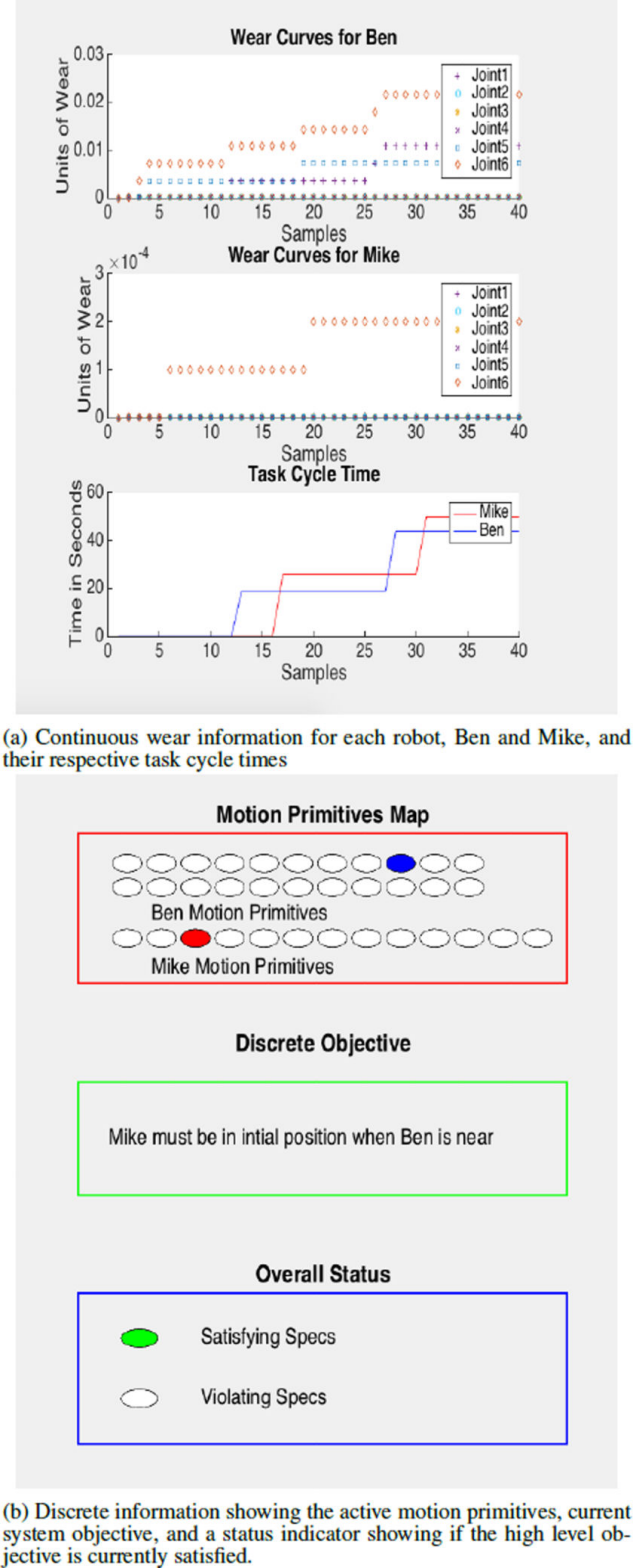
MATLAB interfaces for the continuous and discrete pieces of the monitoring framework

Figure 3 depicts the three dimensional model of the robotic work-cell in the VREP simulation environment. Figure 4 shows the MATLAB interface displaying continuous time wear parameters and the cycle time associated with the two robots along with the discrete system information. In the top figure, continuous information for the whole system has been presented. This includes wear information of all the joints of the robots according to the dynamic functions defined in the previous section. For each of the robots, wear has been computed for all of the six joints. It can be observed that wear curves for robot Ben are more evenly spread on to all the joints. In comparison, wear curves for robot Mike are mostly defined by joint 6. The third graph in Figure 4(a) represents the cycle time for each task that Mike and Ben are performing.

Figure 4(b) conveys information of the system’s discrete nature. The *Motion Primitives* section indicates the current motion primitive of Ben and Mike by filling the corresponding circle for the motion primitive. *Discrete Objective* states the high level overall objective of the system. *Overall Status* indicates if the high level objective specifications are *satisfied* or *violated* by toggling the color of the corresponding bubble.

**Figure 5:**
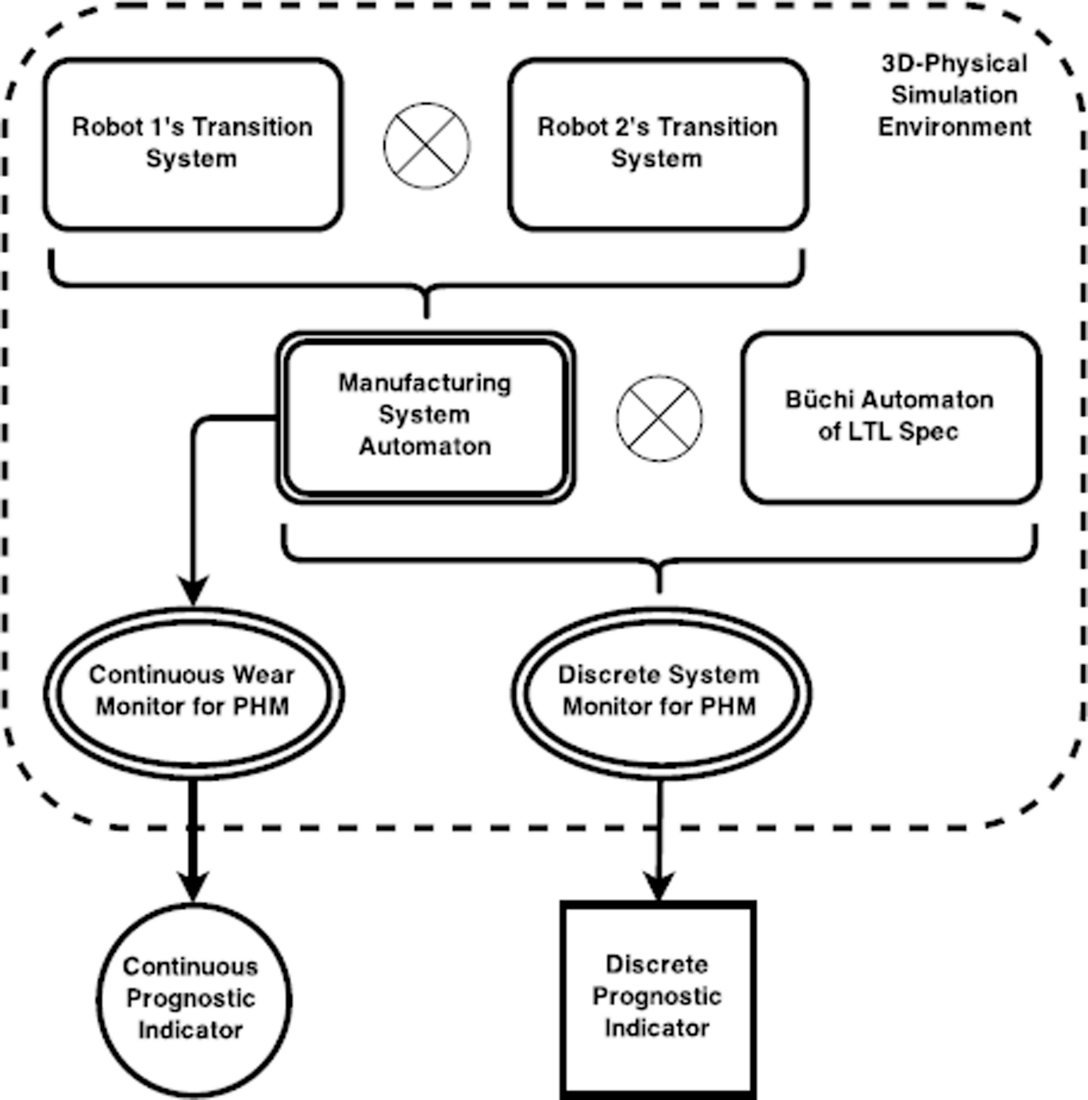
A more general representation of the LTL based monitoring system applied to the use-case where two robots are working together to accomplish a task.

A generalizable structure of the work is defined by Figure 5. The figure is specifically for the use-case where we have two robots that collaborate with each other, but could be extended to include any number of Robotic Transition Systems. The Robotic Transition Systems, which abstract the physical robots present on the factory floor, are subsequently transformed into the overall Manufacturing System Automaton. The plant maintenance team or robotics engineers create the high-level LTL specifications to monitor the discrete exceptions of the Manufacturing System, which is then mathematically written as the Büchi Automaton of the LTL Spec. The LTL Spec and Manufacturing System Automaton can then be represented in the same automaton, which finally becomes the Discrete System Monitor for PHM applications. The actuator wear is also projected for each joint with respect to the robotic systems to monitor continuous parameters. Discrete and Continuous Prognostic Indicators are finally realized, which is exemplified by the MATLAB interface in Figure 4.

### 5.2. Application to Adaptive Multi-Scale PHM

As previously stated, this paper is a part of a larger effort to create an adaptive multi-scale PHM scheme described in (Choo, Beling, LaViers, Marvel, & Weiss, 2015). Adaptive multi-scale prognostics and health management (AM-PHM) is a methodology designed to support PHM in smart manufacturing systems. AM-PHM is characterized by its incorporation of multi-level, hierarchical relationships and PHM information. AM-PHM utilizes diagnostic and prognostic information regarding the current health of the system and constituent components, and propagates it up the hierarchical structure. By doing so, the AM-PHM methodology creates actionable prognostic and diagnostic information along the manufacturing process hierarchy. This information includes the predicted health state upon completion of a task. The health estimates that flow up the hierarchy are based upon simulated operational directives that flow down it. Nodes at given levels along the system hierarchy consume operational profiles from adjacent, higher level nodes. These profiles describe the production goals under consideration by the decision makers (e.g., operators and supervisors) in the superior level. In addition to the traditional workload, bill of materials, and requirements of the manufacturing process, the operational profile may have a focused objective such as minimizing cost or maximizing reliability. Each AM-PHM module creates operational profiles for its subordinate AM-PHM modules while producing diagnostic and prognostic information for its higher level subsystem.

The simulator framework described in this paper would provide the capability to estimate wear and other measures of system health with respect to given operational profiles, and so could be the basis for upward push of prognostics and health estimates. In an attempt to deliver true adaptable and scalable information for translating operational profiles into operational directives, LTL specifications can be hierarchical in nature to capture subtopic levels, or the individual motors, and head topic levels, which is the team process flow.

## 6. Conclusion

The paradigm shift in Advanced Manufacturing, where manufacturers are introducing the next generation of flexible and collaborative robotics, has the potential to further shape the sector. This shift, along with Prognostic and Health Management techniques, is a large part of what will enable Smart Manufacturing Systems. The novel LTL-based monitor reviewed in this work introduces a method for connecting continuous and discrete prognostics, and is immediately applicable to the robotic platforms that manufacturers seek to install in their factories.

We have applied this monitor to an industry-inspired use-case and showed in a three dimensional simulation environment how the methodology can be integrated on a robotic work-cell. The differential wear functions can be installed to fit the manufacturer specific application, and handled by the automated computing environment for generating wear diagnostics. Intuitive high-level specifications can be applied by systems integrators or plant supervisors for filtering out discrete exceptions. This is especially important as production lines in the advanced manufacturing setting employ an increasing suite of sensors to observe their processes.

Therefore, we have laid the ground work for building intelligent control strategies to evenly spread wear of robotic platforms, ergo maximizing the life of the system. Future work will leverage the supervisory control and model checking found in the monitor to define the multiple ways motions can be performed, and then switch between styles of motion to best extend asset life. This automated flexibility continues to close the gap on waste, both in the form of time and capital expenditure.

The LTL-monitor serves as a blueprint for implementing PHM in robotics and all other forms of automation. The protocols can be written to allow for information flow into the larger supply chain systems scheme, further bolstering the Adaptive, Multi-scale PHM environment. The overall vision gives plant leadership teams and operations management alike the structure to seamlessly integrate their manufacturing capabilities with market demand. As pressures for profitability continue, this will undoubtedly be of interest to industry to ensure productivity, quality, and safety goals.
